# Early Biohumoral Detection of Acute Kidney Injury After Robotic Renal Surgery and Its Impact on Medium-Term Renal Function

**DOI:** 10.3390/ijms27083515

**Published:** 2026-04-14

**Authors:** Raffaele La Mura, Alessio Paladini, Paolo Mangione, Guido Massa, Jessica Pagnotta, Federico Ricci, Matteo Mearini, Giuseppe Giardino, Andrea Vitale, Ettore Mearini, Giovanni Cochetti

**Affiliations:** Department of Medicine and Surgery, Urology Clinic, University of Perugia, 06129 Perugia, Italy; alessiopaladini89@gmail.com (A.P.); paolo.mangione@specializzandi.unipg.it (P.M.); guidomassa19911010@gmail.com (G.M.); jessica.pagnotta@specializzandi.unipg.it (J.P.); federico.ricci@specializzandi.unipg.it (F.R.); matteo.mearini@specializzandi.unipg.it (M.M.); giuseppe.giardino@specializzandi.unipg.it (G.G.); andrea.vitale@ospedale.perugia.it (A.V.); ettore.mearini@unipg.it (E.M.); giovanni.cochetti@unipg.it (G.C.)

**Keywords:** acute kidney injury, partial nephrectomy, radical nephrectomy, renal cell carcinoma, β2-microglobulin, cystatin C, interleukin-6, biomarkers, robotic surgery

## Abstract

Renal surgery for localized renal cell carcinoma carries substantial risk of acute kidney injury (AKI) regardless of surgical approach. This prospective study evaluated early biohumoral markers for AKI detection after robotic renal surgery and assessed their prognostic value for 12-month functional outcomes. Adults undergoing robotic renal tumor surgery with a healthy contralateral kidney were enrolled; AKI followed KDIGO 2012 criteria. Biomarkers measured at baseline and 2/24/72 h were serum β2-microglobulin (sβ2) serum IL-6, as well as urinary β2-microglobulin (uβ2), cystatin C (uC), and α2-macroglobulin (uα2M). Kidney function at 12 months was staged according to KDOQI criteria. Among 170 patients (35 radical nephrectomy, RN; 135 partial nephrectomy, PN), 33 developed AKI, more frequently after RN (*p* < 0.001); baseline biomarkers levels were similar. sβ2 was significantly higher at 2/24/72 h, and at 2 h, it achieved an AUC of 0.78 (cut-off 0.17: sensitivity 82%, specificity 60%), remaining the earliest independent predictor of AKI (*p* = 0.015). IL-6 differed at 24 h (AUC 0.80), uC at 72 h (AUC 0.73) and uβ2 at 72 h (AUC 0.66). Clinical AKI predicted KDOQI stage progression at 12 months (*p* < 0.001). Bulldog clamps (mean ischemia time 17.2 ± 6.9 min) were not associated with AKI (*p* = 0.99) or with KDOQI stage progression (*p* = 0.54). RN confers a higher AKI risk than PN. sβ2 at 2 h is the earliest actionable marker, complemented by IL-6 (24 h) and uC (72 h); short warm ischemia during robotic PN appears safe. Sequential multimarker assessment may improve recognition of AKI and support timely nephroprotective strategies.

## 1. Introduction

Renal cell carcinoma (RCC) accounts for approximately 3% of adult malignancies, and its incidence continues to rise globally, largely due to the increasing detection of incidental renal masses through modern cross-sectional imaging [1,2,3]. For patients with T1 localized RCC, current European Association of Urology guidelines recommend partial nephrectomy (PN) as the standard surgical approach whenever technically feasible, given its oncologic equivalence to radical nephrectomy (RN) and superior preservation of renal function [4]. The nephron-sparing rationale underlying PN is clinically meaningful: reduced renal functional reserve is strongly associated with an elevated risk of chronic kidney disease (CKD), cardiovascular morbidity, and all-cause mortality [5,6]. Despite its established advantages, PN frequently necessitates temporary renal hilar clamping to facilitate tumor excision and renal reconstruction. Although several studies support that warm ischemia times <25–30 min are generally safe in patients with a healthy contralateral kidney, ischemia–reperfusion injury remains a relevant determinant of postoperative acute kidney injury (AKI) [7,8]. AKI following renal surgery is clinically relevant. Multiple studies have demonstrated its association with increased short-term complications, prolonged hospitalization, and higher long-term risk of CKD progression [9,10]. After RN, the abrupt reduction in renal mass may precipitate AKI even in the absence of ischemia, while after PN, AKI results primarily from a combination of parenchymal loss, ischemia–reperfusion injury, inflammation, hemodynamic fluctuations and perioperative nephrotoxic exposures [11,12]. Despite refinements in robotic surgery and improved nephron-sparing techniques, the incidence of postoperative AKI remains considerable, ranging from 15% to over 40%, depending on definitions and patient selection [13,14]. The timely identification of AKI remains challenging. Urinary and circulating microRNAs have been investigated both as non-invasive tumor biomarkers in RCC and as indicators of kidney injury, making them particularly attractive candidates to study in the perioperative setting, where oncologic and AKI-related signals may overlap [15,16,17]. Traditional markers, such as serum creatinine (sCr) and derived estimated glomerular filtration rate (eGFR), are late and relatively insensitive indicators of early tubular injury, as they may remain unchanged until substantial functional impairment has already occurred. Consequently, interest has shifted toward novel biomarkers capable of detecting subclinical renal injury during its earliest stages, when timely intervention may still mitigate permanent nephron loss [13,18].

Among promising candidates, β2-microglobulin and cystatin C reflect proximal tubular reabsorptive capacity, whereas inflammatory mediators such as interleukin-6 are increasingly recognized as mediators and potential biomarkers of ischemia–reperfusion injury, correlating with the magnitude of renal insult [19,20]. A recent systematic review highlighted that no single biomarker achieves optimal accuracy across the entire postoperative period, suggesting a potential benefit from multimarker panels. Nevertheless, evidence in the context of robotic PN and RN remains scarce, and the temporal dynamics of these biomarkers—particularly in the very early postoperative hours—are insufficiently characterized. With the evolution of robotic platforms and contemporary renoprotective strategies, updated evidence is needed to clarify the temporal dynamics and predictive accuracy of biohumoral markers in the immediate postoperative period [13].

The primary objective of this prospective study was, therefore, to evaluate a panel of biohumoral markers—including serum β2-microglobulin (sβ2), urinary β2-microglobulin (uβ2), urinary cystatin C (uC), serum IL-6 (sIL-6), and urinary α2-macroglobulin (uα2M)—for the early detection of AKI following robotic PN and RN. The secondary objective was to determine whether these biomarkers, along with clinical and surgical variables, predict renal functional outcomes at 12 months, measured through changes in KDOQI staging. By characterizing a multimarker panel at predefined early postoperative time points, this study aims to refine perioperative risk stratification, facilitate prompt recognition of renal injury, and ultimately support more effective nephroprotective strategies in the setting of modern robotic renal surgery.

## 2. Results

### 2.1. Study Cohort and Baseline Characteristics

A total of 170 patients undergoing robotic renal surgery were included: partial nephrectomy (PN) in 135 (79.4%) and radical nephrectomy (RN) in 35 (20.6%). Mean age was 64.0 ± 12.2 years, and 91 patients (53.6%) were male. Hypertension and diabetes were present in 99 (58.2%) and 16 (9.4%) patients, respectively; mean body mass index was 25.6 ± 3.6 kg/m^2^. Smoking history was evenly distributed, with 86 (50.6%) never-smokers and 84 (49.4%) current or former smokers. Mean tumor diameter was 43.2 ± 24.8 mm; clinical stage distribution was cT1a 60.6%, cT1b 22.9%, cT2 5.9% (T2a 5.3%, T2b 0.6%), and cT3a 10.6%. Final histology was predominantly clear cell renal cell carcinoma (58.2%), followed by papillary (14.1%) and chromophobe (5.9%) RCC, whereas 21.8% of lesions were benign. Baseline renal function was preserved in all patients, with 170/170 (100%) classified as KDOQI stage 1–2. With respect to ischemia strategy, PN was performed without hilar clamping in 58/170 (34.1%), whereas 77/170 (45.3%) underwent PN under warm ischemia using a bulldog clamp; RN accounted for 35/170 (20.6%). Among clamped PN cases, the mean warm ischemia time was 17.2 ± 6.9 min (Table 1).

### 2.2. Incidence of Postoperative AKI and Association with Surgical Approach

Postoperative AKI (KDIGO-defined) occurred in 33/170 patients (19.4%). RN was significantly associated with a higher incidence of AKI compared with PN (Pearson χ^2^ = 24.0, *p* < 0.001) (Table 2).

### 2.3. Ischemia Strategy and Risk of Postoperative AKI

Within the PN cohort, the use of hilar clamping with bulldog clamps and warm ischemia duration (mean 17.2 ± 6.9 min among clamped PN) were not associated with the occurrence of postoperative AKI (Pearson χ^2^ = 0.007, *p* = 0.94) (Table 3).

### 2.4. Biomarker Profiling in Patients with Versus Without AKI

When stratified by AKI status, sβ2 displayed the earliest and most consistent signal: sβ2 was significantly higher in patients developing AKI as early as 2 h (*p* < 0.001), with persistence at 24 and 72 h (both *p* < 0.001). sIL-6 differentiated AKI from non-AKI most prominently at 24 h (*p* = 0.015). Tubular biomarkers showed a delayed but significant separation at 72 h: uC (*p* = 0.008) and uβ2 (*p* = 0.019), whereas uα2M did not differ at any time point (Table 4).

**Figure 1:**
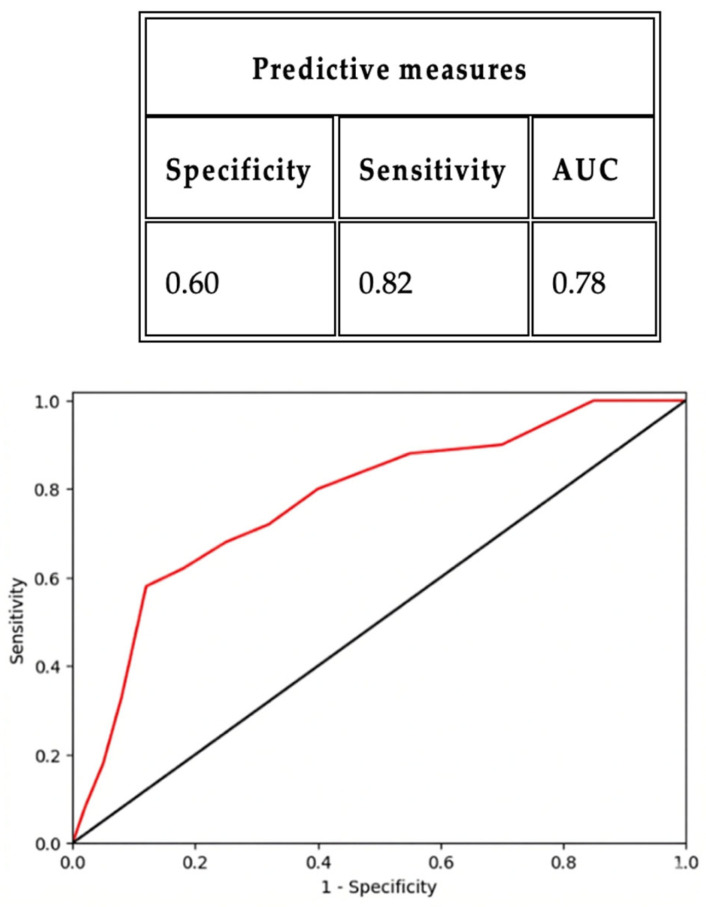
ROC analysis curve to predict AKI event for sβ2 at 2 h (cut-off: 0.17; threshold 0.17 mg/dL).

**Figure 2:**
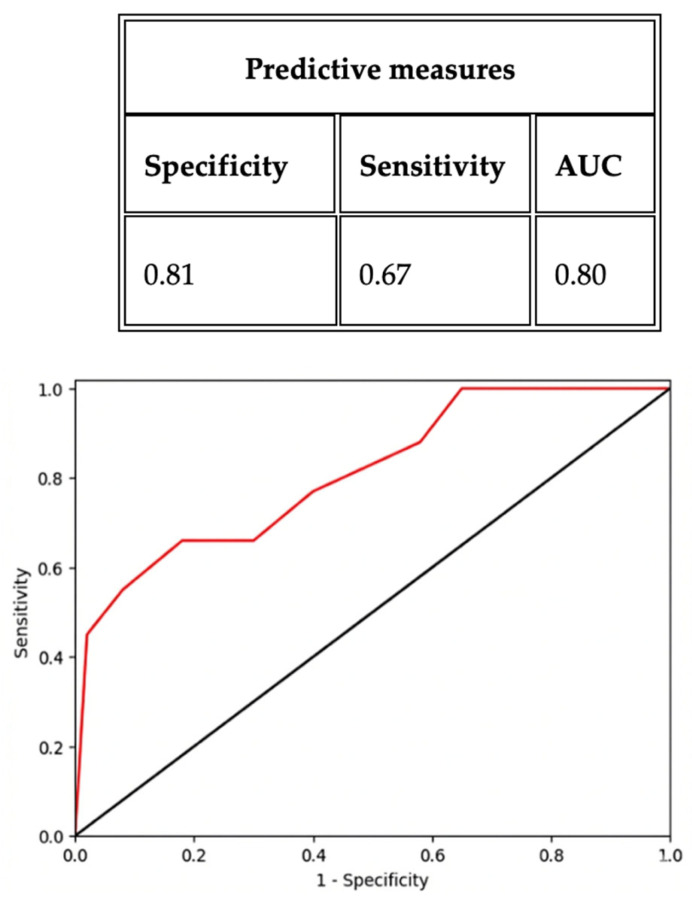
ROC analysis curve to predict AKI event for sIL-6 at 24 h (cut-off: 0.35; threshold 43.3 pg/mL).

**Figure 3:**
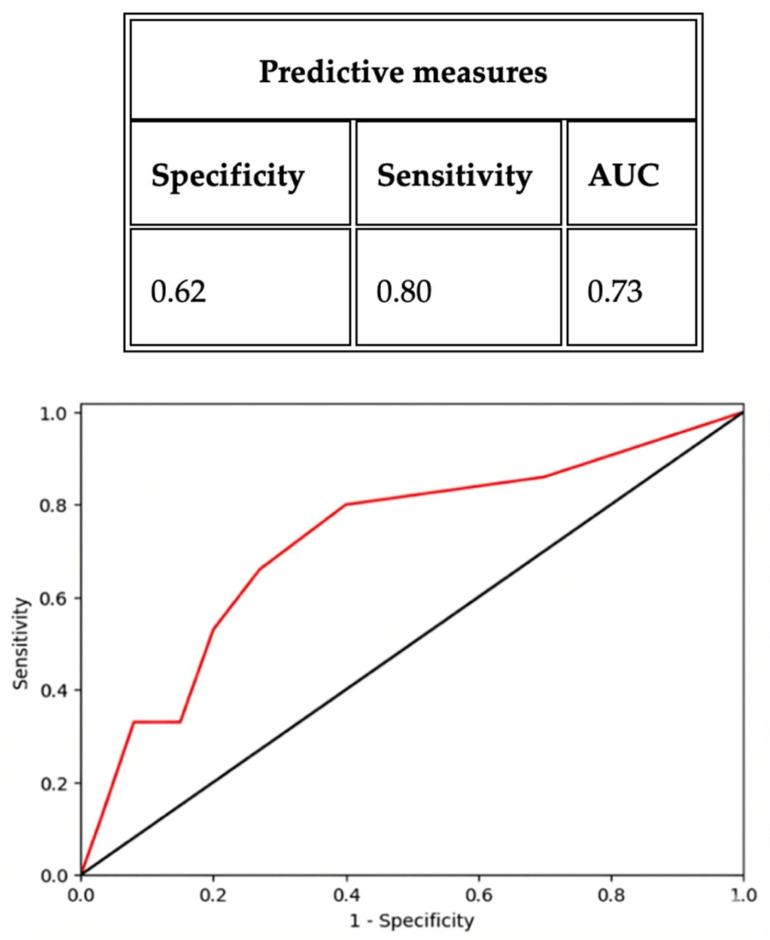
ROC analysis curve to predict AKI event for uC at 72 h (cut-off: 0.17; threshold 0.61 mg/L).

ROC analyses confirmed this time-dependent diagnostic hierarchy: sβ2 at 2 h achieved an AUC of 0.78 (95% CI 0.68–0.88), with a sensitivity of 0.82 and specificity of 0.60 at the optimal threshold defined by the Youden index; sIL-6 at 24 h yielded an AUC of 0.80 (95% CI 0.70–0.90), with a sensitivity of 0.67 and specificity of 0.81 at the Youden index-derived cut-off; and uC at 72 h showed an AUC of 0.73 (95% CI 0.63–0.83), with a sensitivity of 0.80 and specificity of 0.62 at the Youden index-optimized threshold (Figure 1, Figure 2 and Figure 3).

### 2.5. Multivariable Predictors of Postoperative AKI

In multivariable logistic regression, biomarker signals retained independent association with AKI. Specifically, sβ2 at 2 h (per 1 SD increase) independently predicted AKI (OR 1.57, 95% CI 1.10–2.24; *p* = 0.015), as did sIL-6 at 24 h (OR 1.70, 95% CI 1.06–2.73; *p* = 0.028) and uC at 72 h (OR 2.03, 95% CI 1.05–3.92; *p* = 0.036). After adjustment, RN versus PN was not independently associated with AKI (OR 0.77, 95% CI 0.13–4.54; *p* = 0.778) (Table 5).

### 2.6. Medium-Term Renal Functional Outcome (12 Months)

At 12 months, 39/170 patients (22.9%) experienced renal functional deterioration defined as an increase of at least one KDOQI stage. Patients with KDOQI worsening had significantly lower eGFR at 24 h (55.8 ± 21.7 vs. 70.7 ± 21.3 mL/min/1.73 m^2^; *p* = 0.04) and 72 h (60.7 ± 20.2 vs. 74.3 ± 20.6 mL/min/1.73 m^2^; *p* = 0.011), whereas serum creatinine at 24 h showed a borderline association (1.28 ± 0.47 vs. 1.11 ± 0.51 mg/dL; *p* = 0.055). Among biomarkers, sβ2 differed significantly at 2 h, being higher in the KDOQI progression group (KDOQI+) (*p* = 0.047), whereas no significant differences were detected at baseline, 24 h, or 72 h (Table 6).

In the multivariable model for 12-month KDOQI progression, clinical AKI was the only independent predictor of medium-term renal functional deterioration (OR 5.42, 95% CI 1.63–18.0; *p* = 0.006), whereas neither surgical approach nor dichotomized sβ2 remained significant (Table 7).

### 2.7. Ischemia Strategy and 12-Month Renal Functional Outcome

Warm ischemia duration was not associated with 12-month KDOQI stage progression (Pearson χ^2^ = 0.50, *p* = 0.48) (Table 8).

### 2.8. Summary of Findings

In this prospective cohort of patients undergoing robotic renal surgery, we demonstrated that:

- RN is associated with a markedly higher incidence of postoperative AKI than PN, despite the presence of a healthy contralateral kidney.
- Among the panel of biomarkers tested, serum β2-microglobulin at 2 h postoperatively is the earliest independent predictor of AKI, with good diagnostic performance (AUC 0.78, sensitivity 82%, specificity 60% at a cut-off of 0.17 mg/dL).
- sIL-6 at 24 h and uC and uβ2 at 72 h provide complementary information with good-to-moderate diagnostic accuracy, reflecting the inflammatory and tubular injury components of AKI.
- Clinically defined AKI but not the surgical approach or ischemia duration within approximately 20 min independently predicts medium-term deterioration of renal function at 12 months (OR 5.42, 95% CI 1.63–18.0).

## 3. Discussion

Loss of functioning nephrons is a well-established driver of chronic renal failure after RN, and multiple studies have shown that even modest reductions in renal reserve significantly increase the long-term risk of cardiovascular morbidity and mortality [21]. Go et al. followed over one million individuals for 2.8 years and demonstrated a clear stepwise increase in cardiovascular events and all-cause mortality with declining eGFR, independent of hypertension, diabetes, and other confounders [22]. These findings underscore the clinical value of nephron preservation and justify the widespread preference for PN whenever technically and oncologically feasible.

In this context, early detection of perioperative renal injury is of central importance. Several studies have linked postoperative AKI with accelerated renal functional decline, and Martini et al. demonstrated that AKI after robotic PN predicts a significant reduction in glomerular filtration between 3 and 15 months, independently of preoperative renal status [10]. The same group also highlighted modifiable surgical and perioperative factors—such as ischemia duration and type of intravenous fluid—as potential contributors to AKI risk [11]. However, in our cohort, the mean warm ischemia time remained well within the accepted safety window (<25–30 min), and neither ischemia duration nor clamp usage emerged as predictors of AKI or KDOQI progression. This is consistent with the literature suggesting that ischemia under 25–30 min is generally well tolerated in patients with a healthy contralateral kidney [23,24].

The incidence of AKI varies widely across reports, ranging from 0.8% to 54% depending on population, definitions, and timing of assessment. Kara et al. reported a 5.1% incidence of AKI and identified prolonged ischemia and elevated BMI as independent risk factors [12], although such associations were not observed in our study. Allinovi et al. described AKI as a multifactorial perioperative event involving ischemia–reperfusion injury, inflammation, microembolization, endothelial damage, hypotension, anesthetic factors, pneumoperitoneum, hemodilution, and nephrotoxins [25]. Our findings align with this concept, as many of these factors may contribute small but cumulative insults that vary among patients.

Accordingly, recent nephrology literature emphasizes a biological continuum of AKI, in which early stress and damage markers precede functional impairment and may better capture interindividual susceptibility to renal injury in the perioperative setting [26].

Regarding biomarkers, Antonelli et al. proposed serum cystatin C as a promising early marker of tubular dysfunction, rising earlier than serum creatinine and strongly correlating with postoperative AKI and 12-month renal function [13]. However, in our study, urinary cystatin C did not share these characteristics; instead, it distinguished AKI from non-AKI only at 72 h, indicating its utility primarily as a later marker of persistent tubular injury. Chung et al. similarly found no consistent correlation between urinary cystatin C and β2-microglobulin [27]. In contrast, our findings identify serum β2-microglobulin (sβ2) as a clinically meaningful and uniquely early biomarker.

Indeed, the most distinctive result of this investigation is the demonstration that sβ2 rises significantly within two hours after surgery in patients who subsequently develop AKI—well before traditional markers such as serum creatinine and earlier than any other biomarker tested. This rapid increase likely reflects proximal tubular dysfunction induced by ischemia–reperfusion injury, hemodynamic fluctuations, and early inflammatory activation—mechanisms widely described in renal injury models [14,19,28]. While β2-microglobulin has long been recognized as a marker of tubular injury, its ability to discriminate postoperative AKI within such an early window has not previously been demonstrated in robotic nephron-sparing surgery [29].

sIL-6, a mediator of systemic and intrarenal inflammation, distinguished AKI from non-AKI patients at 24 h, consistent with the inflammatory phase of ischemic and surgical injury [30]. The uβ2 and uC showed discriminatory capacity at 72 h, capturing sustained tubular damage rather than the earliest postoperative insult. Conversely, uα2M did not demonstrate diagnostic utility, likely due to its high molecular weight and limited sensitivity to proximal tubular stress [31].

The significantly higher incidence of AKI after RN compared with PN mirrors previous reports and reflects the abrupt parenchymal loss and reduced adaptive capacity associated with RN [32]. However, when sβ2 measured at two hours was incorporated into multivariate analysis, the type of surgery no longer independently predicted AKI. This finding suggests that the mechanistic pathway linking surgical modality to long-term renal decline is mediated primarily through the development of AKI itself rather than nephron loss per se. This observation is consistent with studies emphasizing that the occurrence—and particularly the persistence—of AKI drives medium- and long-term renal dysfunction [33,34].

Our results align with prior literature examining AKI prevalence and impact. Bravi et al. reported AKI rates of 20–30% after PN depending on surgical approach [35], and Makėvičius et al. observed a 42% AKI rate at 48 h, though only 23% met the criteria for clinically significant AKI [14], findings that parallel our cohort. In single-kidney patients, Garofalo et al. showed that postoperative AKI confers a three-fold higher risk of CKD progression [36]. Cho et al. previously documented a 33.7% AKI rate after RN using the RIFLE criteria [37]. In contrast, Zabell et al. argued that surgical injury alone contributes minimally to long-term renal decline and that systemic comorbidities are the primary drivers [33]. However, Bravi et al. demonstrated a clear relationship between postoperative AKI and long-term renal deterioration, emphasizing the prognostic significance of AKI persistence beyond 48–72 h [34]. Our findings support this latter interpretation: AKI emerged as the sole independent predictor of KDOQI progression at 12 months, with an approximately five-fold higher risk of functional decline.

Notably, randomized interventional studies have shown that biomarker-guided identification of high-risk surgical patients can be coupled with standardized perioperative kidney-protective care bundles (e.g., KDIGO-based measures), leading to a reduction in the incidence of postoperative AKI, particularly moderate-to-severe AKI, compared with usual care, thereby supporting the clinical relevance of very-early biomarker signals [38,39].

Taken together, our results indicate that early identification of subclinical renal injury is essential. sβ2 at two hours provides an actionable biomarker for rapid risk stratification, enabling earlier nephroprotective interventions, such as hemodynamic optimization, stricter avoidance of nephrotoxins, and timely nephrology involvement. Later biomarkers—including sIL-6, uC, and uβ2—may refine postoperative monitoring by identifying patients with sustained tubular injury who require closer follow-up. The integration of early, multimodal biomarker monitoring could, therefore, shift perioperative management from reactive to proactive, potentially reducing AKI incidence and attenuating the progression from acute renal insult to chronic kidney disease.

### Strengths and Limitations

The strengths of this study include its prospective design, standardized biomarker collection at predefined postoperative time points, and the homogeneous use of robotic techniques performed by experienced surgeons. The focus on patients with a healthy contralateral kidney minimizes confounding due to pre-existing renal impairment and allows clearer attribution of renal changes to perioperative insult.

Limitations must also be acknowledged. This is a single-center study with a moderate sample size, particularly in the RN subgroup, which may limit generalizability and the precision of certain estimates. Urinary output criteria of the KDIGO definition could not be applied uniformly, potentially leading to underdiagnosis of milder AKI episodes. Additionally, although the biomarker panel captured key aspects of proximal tubular dysfunction and systemic inflammation, other relevant markers such as NGAL, KIM-1, or TIMP-2·IGFBP7 were not assessed and could complement or enhance the predictive model. Finally, while strong associations were observed, interventional trials are needed to determine whether biomarker-guided management strategies can improve clinical outcomes.

## 4. Materials and Methods

### 4.1. Study Design and Population

This prospective, single-center observational study included adult patients (≥18 years) undergoing robotic PN or RN for renal tumors between 2021 and 2024. The study protocol was approved by the local Ethics Committee (CEAS N. 3193/18), and all patients provided written informed consent prior to enrolment. Eligibility criteria required the presence of a radiologically and functionally normal contralateral kidney. Exclusion criteria comprised: solitary functional kidney; known glomerular, interstitial, or vascular renal disease; significant atherosclerotic reno-arterial plaques; active urinary tract infection within 30 days preoperatively; nephrolithiasis; uncontrolled malignancy; cognitive inability to provide informed consent; and loss to follow-up prior to the 12-month assessment. Preoperative data included demographic characteristics, comorbidities, BMI, tumor laterality and size, baseline renal function assessed by serum creatinine and estimated glomerular filtration rate (eGFR) using the CKD-EPI equation, which were recorded prospectively in a dedicated database.

### 4.2. Surgical Technique and Perioperative Management

All surgical procedures were performed robotically using the da Vinci Xi Surgical System (Intuitive Surgical, Inc., Sunnyvale, CA, USA) by experienced urologic surgeons according to standardized institutional protocols. The choice between PN and RN was based on tumor size, location, anatomical complexity, patient comorbidity, and surgeon judgement in accordance with current guidelines. For PN, selective hilar dissection was performed, and warm ischemia was applied using Bulldog vascular clamps when indicated. Ischemia time was recorded from clamp application to release, and renorrhaphy was completed using surgeon-preferred reconstructive techniques. RN consisted of ligation and division of the renal artery and vein without temporary vascular occlusion or parenchymal reconstruction. Perioperative management followed enhanced recovery pathways, with particular attention to optimizing hemodynamics, minimizing nephrotoxic medication exposure, and avoiding perioperative hypotension.

### 4.3. Clinical and Laboratory Assessments

Baseline evaluations included serum creatinine, eGFR, and urinalysis. Serum creatinine, blood urea nitrogen, and urine creatinine were measured at baseline and at 2, 24, and 72 h postoperatively. Renal function at 12 months was reassessed through serum creatinine, eGFR, and KDOQI staging.

### 4.4. Biomarker Sampling and Assays

Blood and urine samples were collected at four predefined time points:

1. Baseline: within 24 h before surgery;
2. 2 h postoperatively;
3. 24 h postoperatively;
4. 72 h postoperatively.

At each time point, the following biomarkers were measured:

- Serum β2-microglobulin (sβ2);
- Urinary β2-microglobulin (uβ2);
- Urinary cystatin C (uC);
- Serum interleukin-6 (sIL-6);
- Urinary α2-macroglobulin (uα2M).

Samples were centrifuged, processed, and stored at −80 °C until analysis. Immunoturbidimetric assays (Tina-quant^®^ β2-Microglobulin on the cobas c 501 module, Roche Diagnostics GmbH, Mannheim, Germany) were used for sβ2 and uβ2; cystatin C and uα2M were quantified using ELISA kits (Human Cystatin C Quantikine ELISA Kit, R&D Systems, Inc., Minneapolis, MN, USA; and Human alpha 2 Macroglobulin ELISA Kit, Cambridge, UK) validated for research applications, and sIL-6 was measured using an ultra-sensitivity digital immunoassay (Simoa^®^ IL-6 Advantage PLUS Assay on the HD-X Analyzer, Quanterix Corporation, Billerica, MA, USA). All assays were performed in duplicate by laboratory personnel blinded to the clinical outcomes.

### 4.5. Definitions and Follow-Up

Postoperative AKI was defined according to the KDIGO 2012 criteria using changes in serum creatinine after surgery. Urine output criteria were applied when complete hourly measurements were available; however, creatinine-based definitions served as the standard across the cohort. Patients were stratified into AKI and non-AKI groups. The primary endpoint was the occurrence of postoperative AKI. The secondary endpoint was renal function at 12 months, expressed as KDOQI stage and change in eGFR relative to baseline. Progression was defined as an increase of ≥1 KDOQI stage. Patients without 12-month follow-up data were excluded from the secondary analysis.

### 4.6. Statistical Analysis

Continuous variables were tested for normality using the Shapiro–Wilk test and presented as mean ± standard deviation or median and interquartile range, as appropriate. Categorical variables were summarized as frequencies and percentages. Between-group comparisons (AKI vs. non-AKI; PN vs. RN) were performed using the Student’s *t*-test or Mann–Whitney U test for continuous variables and χ^2^ or Fisher’s exact test for categorical variables. The diagnostic accuracy of each biomarker at each time point was assessed using receiver-operating-characteristic (ROC) curve analysis. Area under the curve (AUC) values and 95% confidence intervals were computed. Optimal cut-offs were determined using Youden’s index. Multivariable logistic regression models were constructed to identify independent predictors of postoperative AKI. Biomarkers were dichotomized based on ROC-derived cut-offs. A second multivariable model evaluated predictors of 12-month KDOQI stage progression, including AKI occurrence, intervention type (PN vs. RN), ischemia time, and biomarker values. Statistical significance was set at *p* ≤ 0.05. Analyses were conducted using SPSS version 26 (IBM Corp., Armonk, NY, USA).

## 5. Conclusions

sβ2 measured 2 h after robotic renal surgery represents the earliest independent biomarker of postoperative AKI among those assessed in this study, providing a clinically meaningful signal before the later changes observed for sIL-6 and uC. Although RN was associated with a significantly higher incidence of AKI, postoperative AKI—rather than surgical approach or warm ischemia duration per se—was the sole independent predictor of renal function deterioration at 12 months. These findings support the potential role of early multimarker biohumoral monitoring, with sβ2 as a key component, in improving postoperative risk stratification and supporting timely nephroprotective management after robotic renal surgery.

### Future Perspectives

Future research in robotic renal surgery should move beyond the diagnosis of postoperative AKI and focus on procedure-specific perioperative risk stratification. In this setting, early biomarkers such as sβ2 may be integrated with baseline renal function, tumor size and complexity, and surgical variables to identify patients at highest risk after surgery. Combining early biomarker profiling with side-specific functional assessment may further clarify the relative contribution of parenchymal loss, ischemia, and compensatory contralateral adaptation to postoperative renal outcomes. Larger multicenter studies are needed to validate these findings across broader patient populations, including those with baseline CKD or solitary kidneys. Prospective studies should also evaluate whether biomarker-guided nephroprotective strategies—such as tailored hemodynamic optimization, stricter avoidance of nephrotoxins, and early nephrology involvement—can reduce AKI incidence and improve medium- and long-term renal functional outcomes after robotic partial and radical nephrectomy.

## Figures and Tables

**Table 1:** Socio-demographic characteristics of enrolled patients (*n* = 170).

Variable	Value
Age, mean ± SD	64.0 ± 12.2
Sex, *n* (%)	
Men	91 (53.5%)
Women	79 (46.5%)
BMI, mean ± SD	25.6 ± 3.6
Diabetes, *n* (%)	16 (9.4%)
Hypertension, *n* (%)	99 (58.2%)
Smoking status, *n* (%)	
Never	86 (50.6%)
Current or Former	84 (49.4%)
Tumor diameter at imaging (mm), mean ± SD	43.2 ± 24.8
Clinical T stage, *n* (%)	
T1a	103 (60.6%)
T1b	39 (22.9%)
T2a	9 (5.3%)
T2b	1 (0.6%)
T3a	18 (10.6%)
Type of intervention and ischemia strategy, *n* (%)	
PN—No ischemia	58 (34.1%)
PN—Bulldog clamp	77 (45.3%)
RN	35 (20.6%)
Ischemia time, min (excluding RN and no-ischemia PN), mean ± SD	17.2 ± 6.9
Histology, *n* (%)	
Benign	37 (21.8%)
Clear cell RCC	99 (58.2%)
Papillary RCC	24 (14.1%)
Chromophobe RCC	10 (5.9%)
Grading, *n* (%)	
Not applicable	42 (24.7%)
G1	19 (11.2%)
G2	89 (52.4%)
G3	13 (7.6%)
G4	7 (4.1%)
Baseline KDOQI stage, *n* (%)	
Stage 1	58 (34.1%)
Stage 2	112 (65.9%)
Clinical AKI, *n* (%)	33 (19.4%)
Increase in KDOQI stage at 12 months, *n* (%)	39 (22.9%)

**Table 2:** Association between surgical approach and postoperative AKI.

Clinical AKI (KDIGO)	PN	RN	Total
No	119	18	137
Yes	16	17	33
Total	135	35	170

**Table 3:** Association between ischemia strategy and postoperative AKI.

Clinical AKI (KDIGO)	Warm Ischemia	No-Ischemia	Total
No	68	51	119
Yes	9	7	16
Total	77	58	135

**Table 4:** Baseline, 2 h, 24 h, and 72 h laboratory results among patients who experienced (33) vs. those who did not experience (137) AKI.

	Baseline			2 h			24 h			72 h		
	Non-AKI	AKI	*p*	Non-AKI	AKI	*p*	Non-AKI	AKI	*p*	Non-AKI	AKI	*p*
sβ2 (mg/dL)	0.18 (0.1–0.66)	0.215 (0.12–0.84)	0.084	0.17 (0.05–0.75)	0.28 (0.13–0.9)	<0.001	0.20 (0.09–0.49)	0.30 (0.15–0.96)	<0.001	0.19 (0.1–0.83)	0.285 (0.16–1.31)	<0.001
uβ2 (mg/L)	0.17 (0.17–2.59)	0.17 (0.17–0.69)	0.477	0.295 (0.17–6.83)	0.20 (0.17–10.8)	0.643	0.26 (0.17–37.9)	0.19 (0.14–16.7)	0.305	0.43 (0.17–24.7)	1.36 (0.17–19.4)	0.019
uC (mg/L)	0.08 (0–0.24)	0.1 (0–0.24)	0.705	0.10 (0–0.39)	0.09 (0–0.25)	0.617	0.21 (0–0.88)	0.28 (0.03–0.36)	0.259	0.05 (0–0.30)	0.22 (0–0.46)	0.008
uα2M (mg/L)	3 (3–3)	3 (3–3)	0.846	3 (3–8.3)	3 (3–6.5)	0.726	3 (0–60)	3 (0–17)	0.621	3 (3–4.5)	4 (3–22.1)	0.238
sIL-6 (pg/mL)	8 (2.3–21.1)	14.2 (2.2–39.6)	0.147	21.5 (11.6–67.3)	65.5 (21.1–246)	0.103	36.3 (10.6–81)	110.3 (10.7–282)	0.015	19.9 (12.7–44.2)	57.8 (30.2–229)	0.069

**Table 5:** Multivariate logistic regression analysis identifying independent predictors of postoperative AKI.

Variable	β (SE)	OR (95% CI)	*p* Value
sβ2 (per 1 SD increase, 2 h)	0.451 (0.185)	1.57 (1.10–2.24)	0.015
sIL-6 (per 1 SD increase, 24 h)	0.528 (0.241)	1.70 (1.06–2.73)	0.028
uC (per 1 SD increase, 72 h)	0.709 (0.338)	2.03 (1.05–3.92)	0.036
RN vs. PN	−0.257 (0.913)	0.77 (0.13–4.54)	0.778

**Table 6:** Baseline, 2 h, 24 h, and 72 h laboratory results among patients who experienced (39) vs. those who did not experience (131) an increase in their KDOQI stage at 12 months.

	Baseline			2 h			24 h			72 h		
	No	KDOQI+	*p*	No	KDOQI+	*p*	No	KDOQI+	*p*	No	KDOQI+	*p*
BUN (mg/dL)	39.3 ± 13.9	43.8 ± 13.3	0.127	39.3 ± 18.7	40.3 ± 12.9	0.436	41.6 ± 17.1	48.6 ± 18.8	0.093	37.9 ± 23.6	39.8 ± 15.8	0.403
Creatinine (mg/dL)	1 ± 0.39	0.93 ± 0.35	0.376	1.1 ± 0.51	1 ± 0.26	0.795	1.11 ± 0.51	1.28 ± 0.47	0.055	1.07 ± 0.66	1.17 ± 0.47	0.254
eGFR (mL/min/1.73 m^2^)	77.3 ± 18.9	76.8 ± 19.1	0.99	71.6 ± 21.4	65.6 ± 16.4	0.178	70.7 ± 21.3	55.8 ± 21.7	0.041	74.3 ± 20.6	60.7 ± 20.2	0.011
sβ2 (mg/dL)	0.19 (0.1–0.84)	0.19 (0.13–0.66)	0.971	0.18 (0.05–0.9)	0.29 (0.11–0.75)	0.047	0.20 (0.09–0.96)	0.24 (0.12–0.46)	0.585	0.20 (0.1–1.31)	0.22 (0.11–0.83)	0.122
uβ2 (mg/L)	0.17 (0.17–1.09)	0.17 (0.17–0.72)	0.232	0.29 (0.17–6.83)	0.20 (0.17–10.8)	0.75	0.26 (0.14–37.9)	0.20 (0.14–16.7)	0.814	0.43 (0.17–23.1)	0.77 (0.17–24.7)	0.119
uC (mg/L)	0.08 (0–0.24)	0.1 (0–0.24)	0.779	0.12 (0–0.39)	0.05 (0–0.25)	0.191	0.24 (0–0.88)	0.21 (0–0.36)	0.572	0.06 (0–0.46)	0.06 (0–0.46)	0.507
uα2M (mg/L)	3 (3–3)	3 (3–3)	0.965	3 (3–6.5)	4.3 (3–8.3)	0.126	3 (3–17)	3 (0–60)	0.318	3 (3–22.1)	3.75 (3–4.5)	0.646
sIL-6 (pg/mL)	3.75 (1–39.6)	21.1 (10.4–27.8)	0.549	24.9 (11.6–246)	23.4 (7.6–33.1)	0.754	15.9 (10.6–141)	48.7 (28.2–282)	0.259	23.4 (12.7–55.6)	129.6 (29.9–229)	0.089

**Table 7:** Multivariate logistic regression analysis evaluating predictors of KDOQI stage progression at 12 months.

Predictor	β (SE)	Z	*p* Value	Odds Ratio (95% CI)
Intercept	−0.62 (0.91)	−0.68	0.50	0.54
Clinical AKI	1.69 (0.61)	2.77	0.006	5.42 (1.63–18.0)
sβ2 at 2 h (≥cut-off)	0.41 (0.88)	0.47	0.64	1.51 (0.27–8.52)
RN vs. PN	0.36 (0.67)	0.54	0.59	1.43 (0.38–5.41)

**Table 8:** Association between ischemia strategy and 12-month KDOQI stage progression.

Increased KDOQI Stage (≥1)	Warm Ischemia	No-Ischemia	Total
No	65	50	115
Yes	12	8	20
Total	77	58	135

## Data Availability

The data presented in this study are available on reasonable request from the corresponding author. The data are not publicly available due to privacy.
